# A Prospective Treatment Option for Lysosomal Storage Diseases: CRISPR/Cas9 Gene Editing Technology for Mutation Correction in Induced Pluripotent Stem Cells

**DOI:** 10.3390/diseases5010006

**Published:** 2017-02-24

**Authors:** Chloe L. Christensen, Francis Y. M. Choy

**Affiliations:** Department of Biology, Centre for Biomedical Research, University of Victoria, 3800 Finnerty Rd., Victoria, BC V8P 5C2, Canada; chloechr@uvic.ca

**Keywords:** CRISPR-Cas9, gene editing, lysosomal storage disease, induced pluripotent stem cells, genetic disease

## Abstract

Ease of design, relatively low cost and a multitude of gene-altering capabilities have all led to the adoption of the sophisticated and yet simple gene editing system: clustered regularly interspaced short palindromic repeats/CRISPR-associated protein 9 (CRISPR/Cas9). The CRISPR/Cas9 system holds promise for the correction of deleterious mutations by taking advantage of the homology directed repair pathway and by supplying a correction template to the affected patient’s cells. Currently, this technique is being applied in vitro in human-induced pluripotent stem cells (iPSCs) to correct a variety of severe genetic diseases, but has not as of yet been used in iPSCs derived from patients affected with a lysosomal storage disease (LSD). If adopted into clinical practice, corrected iPSCs derived from cells that originate from the patient themselves could be used for therapeutic amelioration of LSD symptoms without the risks associated with allogeneic stem cell transplantation. CRISPR/Cas9 editing in a patient’s cells would overcome the costly, lifelong process associated with currently available treatment methods, including enzyme replacement and substrate reduction therapies. In this review, the overall utility of the CRISPR/Cas9 gene editing technique for treatment of genetic diseases, the potential for the treatment of LSDs and methods currently employed to increase the efficiency of this re-engineered biological system will be discussed.

## 1. Lysosomal Storage Diseases

Lysosomal storage diseases (LSDs) are a collection of more than 50 severe genetic diseases resulting from deleterious mutations that render lysosomal enzymes, necessary for regulating the endosomal-autophagic-lysosomal system, non-functional [1]. These mutations lead to a cascade of symptoms due to the relentless accumulation of undegraded substrates, macromolecules and metabolites within the lysosome. Each one of the diseases comprising the group of LSDs is considered a rare genetic disease due to a prevalence ranging between 1:57,000 and 1:4,200,000 individuals; however, a combined prevalence across all LSDs indicates a prevalence as high as 1:5000 [2,3]. Notable symptoms presenting across this disease class include hepatosplenomegaly, ischemic stroke, seizures, cardiovascular involvement, and musculoskeletal and neurodegenerative manifestations [4,5,6,7,8]. The most frequently-occurring LSD, Gaucher disease (GD: OMIM 230800, type 1, non-neuronopathic; OMIM 230900, type 2, acute neuronopathic; OMIM 231000, type 3, sub-acute neuronopathic), has an autosomal recessive mode of inheritance, a neurodegenerative component in the most severe forms, and has been shown to exhibit a marked concurrence with Parkinson’s disease (PD) in patients with GD. An investigation into the concurrence of PD and GD by Alcalay and others found that patients with type 1 GD were at a higher risk of developing PD than heterozygotes for *GBA* mutations, and both type 1 GD individuals and *GBA* mutation heterozygotes were at a higher risk of developing PD than non-carriers, although the difference in concurrence between type 1 GD individuals and *GBA* mutation heterozygotes was not statistically significant [9]. Further investigations have identified a genetic link between other rare LSDs, such as Niemann-Pick A disease and neuronal ceroid lipofuscinosis, with a far more commonly occurring disease, PD [10,11,12]. LSDs including GD, Niemann-Pick A disease, Tay-Sachs disease (TSD) and mucolipidosis IV are particularly common in Ashkenazi Jewish populations, showing predicted prevalences as high as 1:640 [13,14,15,16]. In this review, we describe LSDs that are suitable for regenerative therapies utilizing genome editing based on the following criteria: causative mutations are monogenic, target tissues can successfully uptake and utilize supplemental lysosomal enzymes, and current available therapies are limited. Although the vast majority of LSDs fit these criteria, we have chosen Niemann-Pick A disease, Sanfilippo B syndrome, and Pompe disease as typical examples for clarity and brevity.

### 1.1. Current LSD Treatments

Currently, multiple approaches to facilitate the treatment of LSDs are available. These treatment options include enzyme replacement therapy (ERT), substrate reduction therapy (SRT), pharmacological chaperone therapy (PCT) and hematopoietic stem cell transplantation (HSCT), as well as a multitude of treatments used in an attempt to keep secondary effects at bay [17]. None of these aforementioned therapies are currently curative [17]. Treatment of the secondary effects aims to alleviate symptoms associated with particular LSDs and symptoms that are patient-specific, whereas the intent of ERT, SRT and PCT is to target and reduce the accumulation of undegraded substrates within the lysosomes. Where at first, the modification and supplementation of drugs functioning to restore the normal balance of waste reduction in lysosomes appears to be an overarching solution to these diseases, many of these drugs cannot penetrate the protective and nutritive capillary system surrounding the CNS referred to as the blood-brain barrier (BBB), which prevents the passage of large macromolecules to the tissues of the nervous system. For this reason, the severe neurodegenerative pathology is more complicated to treat. Exceptions include two small molecule drugs: ambroxol (used as a PCT for GD) and miglustat (used as a SRT for GD and Niemann-Pick C (NPC) disease), which are capable of crossing the BBB [18,19,20]. Some LSDs are also receptive to a combination therapy, whereby SRT and HSCT acts synergistically to subside disease symptoms [21,22]. LSDs are seen as particularly applicable candidates for treatment with gene-therapy for two major reasons: many of the affected enzymes can be secreted into the surrounding extracellular fluid for uptake via the mannose-6-phosphate (M6P) receptor on diseased cells to act upon and degrade certain target substrates, and the threshold percent enzyme activity necessary to overcome disease symptoms can be quite low [23,24,25].

### 1.2. Drawbacks to Current LSD Treatments

HSCT, the first of the aforementioned treatments available for LSDs, acts as a therapy for patients by supplementing the missing or defective enzyme through donor cells upon a successful engraftment of HSCs. The transmigration of HSCs to visceral organs and to the central nervous system is necessary for these cells to then differentiate into respective, specific cell types [26,27]. Once these cells have differentiated, the enzyme can be released into the extracellular fluids and transferred to affected cells by a process known as cross-correction in order to alleviate symptoms in that tissue [24]. An in depth review demonstrating the utility of HSCT in conjunction with genome editing for potential treatment of LSDs has been described elsewhere [28]. A drawback associated with HSCT is acute or chronic graft-versus-host disease (GVHD) or a rejected engraftment resulting from allogeneic hematopoietic stem cells (HSCs) with an imperfect HLA match. Even in the event that a perfect HLA-matched donor of HSCs is available, certain LSDs do not benefit therapeutically from HSCT. In the case of I-Cell disease, defective *N*-acetylglucosamine-1-phosphate transferase (GlcNAc phosphotransferase) in the Golgi apparatus is unable to phosphorylate mannose residues to M6P, a crucial step in tagging lysosomal enzymes for transport to their destination, the lysosome [29]. Since the production of M6P moieties is diminished in these patient’s cells, many lysosomal enzymes fail to reach the lysosome thus affecting numerous pathways of substrate degradation. The delivery of active GlcNAc phosphotransferase via transplanted HSCs has been speculated to produce limited disease improvement due to the number of pathways affected, and this lack of pathological improvement has been displayed through clinical cases of HSCT in I-Cell disease patients [29].

In addition to HSCT, therapies aimed at replacing defective enzymes, modifying non-functional enzymes, or ‘debulking’ substrate levels have been undertaken. One of these therapies, ERT, has been successfully used to treat LSDs including type 1 GD, Fabry disease, mucopolysaccharidosis I, II, VI and Pompe disease (for review, see [30]). In many cases, ERT is a life-long process that requires patients to receive injections of recombinant enzyme in order to alleviate visceral symptoms associated with their LSD. ERT cannot, however, reduce neurological symptoms associated with these diseases because the exogenous enzyme fails to cross the BBB. LSDs are also suitable targets for gene therapy, which is a form of renewable ERT where a transgene is incorporated into affected cells using viral vectors for long-term expression of that particular gene product. LSDs can utilize the previously described cross-correction mechanism, which allows for the transfer of lysosomal enzymes between neighboring cells [24]. However, these viral gene therapies have their own caveats, such as insertional mutagenesis, transient gene expression, and the development of adaptive immune responses against the introduced viral vector [31,32]. Table 1 summarizes a number of LSDs and the currently available treatment options for patients affected by these diseases.

## 2. iPSCs: Autologous Stem Cell Transplantation

A proposed mechanism to overcome the disadvantages of existing LSD therapies has resulted from a capacity to revert a patient’s terminally differentiated cells to a pluripotent state by using ‘reprogramming’ transcription factors. In a ground-breaking study by Takahashi and Yamanaka, these necessary reprogramming factors were discovered to be c-Myc, Klf4, Oct3/4, and Sox2 (MKOS) [54]. Once reprogrammed, stem cells have a self-renewal capability that renders them useful for regenerative medicine. iPSCs can be used for disease modeling of a myriad of diseases, including LSDs and neurodegenerative disorders associated with LSDs, such as PD, or metabolically similar neurodegenerative disorders, such as NPC disease and Alzheimer’s disease, where both are associated with an accumulation of tau protein tangles [9,55,56,57]. Upon the advent of iPSC technology [54], proof of concept studies quickly emerged [58] demonstrating the therapeutic potential of patient-derived iPSCs for treatment of blood disorders such as sickle cell anemia, and the differentiation of iPSCs to neural progenitor cells for CNS engraftment in humanized mouse models [59]. Additionally, the efficacy of this neural progenitor intracerebral transplantation method has been demonstrated in mouse models of LSDs such as Niemann-Pick A disease, where disease improvement was noted [60]. The studies by Hanna et al. [58] and Wang et al. [59] strengthen the line of reasoning behind the use of iPSCs as a treatment for genetic disease where disease progression affects a host of body systems, such as in many rare LSDs. The use of gene editing technologies, such as the rapidly advancing CRISPR/Cas9 method, is serving to establish a clinically relevant curative stem cell therapy through the correction of underlying disease-causing mutations in patient-derived iPSCs, which can then be differentiated to the desired progenitor cell types for engraftment and cell replacement therapy.

## 3. CRISPR/Cas9 Gene Editing

### 3.1. Current Gene Editing Systems

An elegant and easy-to-use gene editing system, CRISPR/Cas9, is a conceivable, alternative therapeutic option for treating LSDs. This bioengineered tool possesses the ability to selectively target genes containing mutations that lead to non-functional products and correct the disease-causing mutations, in vitro and in vivo [61,62,63,64,65,66,67]. Despite the interest CRISPR/Cas9 has generated regarding the potential of gene editing to correct genetic disease [61,63,66,67,68], this is not the first gene editing tool proposed for this purpose. Three other gene editing platforms have been explored, including zinc finger nucleases (ZFNs), transcription activator-like effector nucleases (TALENs), and homing endonucleases (for reviews, see [69,70]). The utility of these alternative gene editing platforms is lacking in a number of areas where CRISPR/Cas9 excels. For instance, ZFNs are most effective when targeting G rich sequences due to a conferred stability through arginine residues and guanine bases in the major groove, thus limiting the genomic range for this bioengineered tool [71,72]. In addition to other associated design limitations, ZFNs are difficult to design and employ due to the complex and challenging nature of predicting DNA-protein interactions [71]. The CRISPR/Cas9 system makes use of predictable Watson-Crick base pairing, thus allowing for unprecedented ease-of-design.

### 3.2. CRISPR/Cas9: Molecular Design

**Figure 1:**
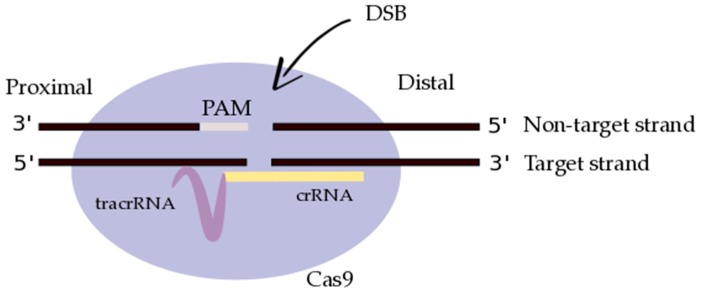
A single guide RNA (gRNA), comprised of CRISPR-derived RNA (crRNA) (purple) and a trans-activating CRISPR RNA (tracrRNA) (yellow), targets Cas9 endonuclease (light purple) to a DNA sequence of interest (black). Cas9 creates a double-stranded break (DSB) in the DNA backbone, instigated by the protospacer-adjacent motif (PAM; light grey) recognition sequence present in the DNA sequence of interest. The DNA strands have been designated as non-target versus target, and proximal versus distal, based on their relative orientation to the gRNA and to the PAM sequence, respectively (adapted from Richardson et al. [81]).

Originally identified as a prokaryotic acquired immune system, CRISPR has undergone certain modifications in order to refine this system as a gene editing tool [73,74,75]. In this CRISPR/Cas9 system, the signature Cas9 protein works in conjunction with two RNAs to home in on a target sequence and successfully introduce double stranded breaks (DSBs) into the DNA backbone of the target DNA. These two RNAs include a CRISPR-derived RNA (crRNA) complimentary to the non-target sequence as well as a trans-activating CRISPR RNA (tracrRNA) that links together the Cas9 endonuclease and the crRNA (Figure 1) [75]. Cas9 employs HNH and RuvC endonuclease domains, wherein the former has been found to be responsible for cleavage of the target strand, and the latter responsible for cleavage of the non-target strand [76,77]. In order for Cas9 to cleave at a target site, a protospacer adjacent motif (PAM) present in the target DNA is necessary to act as a recognition sequence. This PAM sequence is in the form of 5′-NGG-3′ (where ‘N’ is defined as any DNA base) and is contained in the non-target strand [78]. Upon the introduction of a DSB, the inherent cellular repair machinery can follow one of two pathways: the non-homologous end-joining (NHEJ) pathway or the homology directed repair (HDR) pathway, the former inducing insertions and deletions at the site of the break, and the latter allowing for recombination through an area of homology [79,80]. NHEJ is particularly useful for knocking out genes, whereas HDR can be employed with an exogenous correction template to induce specific changes in the DNA sequence at the site of the DSB.

## 4. Proof of Concept Studies: CRISPR/Cas9 for Correction of Genetic Disease

### 4.1. Compound Heterozygous Mutation Correction in β-Thalassemia

Although this technology has not, as of yet, been used for the treatment of LSDs, an abundance of studies have been conducted using CRISPR/Cas9 RNA-guided nucleases (CRISPR/Cas9-RGNs) and HDR to correct disease-associated mutations in human iPSCs and in animal models since 2013 when the technique was adopted [61,62,63,64,66]. One such example is the use of CRISPR/Cas9 and a *piggyBac* construct to correct mutations implicated in β-thalassemia [61]. β-thalassemia is a severe form of anemia resulting from mutations in the β-globin gene *HBB* that leads to a reduction in hemoglobin production. Currently, treatment for β-thalassemia includes repeat blood transfusions, subsequent iron chelation therapy, and, for some, HSCT in cases where a suitable donor is available [61]. Ideally, gene editing in iPSCs derived from the patient, differentiation to multipotent HSCs, and subsequent transplantation back into the patient would overcome the disease state in full. In their study, Xie et al. used Cas9 double strand cleavage at a location flanked by both mutations leading to β-thalassemia in the patient, allowing for HDR to occur between areas of homology within the target sequence and the *piggyBac* correction template [61]. Clones that successfully underwent HDR contained a puromycin and thymidine kinase marker, allowing for selection through the use of puromycin, application of transposase to remove the *piggyBac* transposon upon selection, and thymidine kinase negative selection to eliminate clones that inadvertently integrate the *piggyBac* transposon at other genomic sites [61]. Confirmation of HDR through PCR amplification and Southern blot analysis showed gene correction in 23.5% of the clones, thus indicating a substantial degree of gene editing and repair [61]. A similar approach using a plasmid-based correction template and multiple gRNAs for mutation correction in patients with a compound (bi-allelic) heterozygous genotype would be especially useful in LSDs where cross-correction mechanisms exist.

### 4.2. Mutation Correction in Other Inherited Monogenic Diseases

CRISPR/Cas9 has also been used for the correction of a mutation in the *Crygc* gene, which leads to the generation of cataracts in mice [63]. In contrast to the study by Xie et al. [61], Wu et al. used mouse zygotes for the injection of Cas9 mRNA and a gRNA complimentary to the specified *Crygc* mutation and successfully identified fertile, recombinant mice who transferred the newly corrected allele to their offspring, and in which only a small minority had off-target binding events [59]. Duchenne muscular dystrophy (DMD), an X-linked genetic disorder that results in progressive muscle weakness and a characteristically shortened lifespan, has also been the aim for repair using CRISPR/Cas9 technology in a mouse disease model [64]. DMD occurs in humans at a similar frequency as LSDs, with a rate of 1:3600 to 1:6000 live male births [82]. The specific gene target in the study by Long and colleagues was the *Dmd* gene, in which *mdx* mice harbor a disruptive mutation in exon 23 [64]. Similar to the study by Wu et al. [63], the mouse zygotes were injected with gRNA, Cas9 and the correction template necessary to generate HDR [64]. Long et al. noted that gene editing produced genetically mosaic mice with between 2% and 100% *Dmd* correction and very few off-target effects were noted [64]. Recently, a mouse model of hemophilia B was both created and corrected using CRISPR/Cas9 in vivo, thus demonstrating the success of this gene editing system [66]. These applications of this gene editing technique encompass the breadth of CRISPR/Cas9’s medical relevance through the potential for genetic mutation correction in disease-modeling organisms and are projected methods for correcting mutations associated with genetic disease in humans.

## 5. Clinical Potential, Prospective Applications, and Challenges Using CRISPR/Cas9

### 5.1. CRISPR/Cas9 in Clinical Trials

As previously mentioned, a curative option for patients with LSD is that of CRISPR/Cas9-RGNs for the restoration of enzyme activity in autologous, patient-derived cells. For somatic-cell applications, terminally differentiated cells can be harvested from the patient, cultured, and reprogrammed to iPSCs via the aforementioned pathway described in Takahashi and Yamanaka’s work on reprogramming terminally differentiated cells to iPSCs [54]. Clinical trials have not yet commenced for the treatment of genetic disease using CRISPR/Cas9-RGNs; however, phase I clinical trials, where T cells are re-engineered to combat lethal diseases, have begun. Although the use of CRISPR/Cas9-RGNs is still in the pre-clinical and early clinical stages, the modification of human cells using other gene editing platforms has made its way to clinical applications in recent years. One such study by Perez et al. nullified M-tropic strain HIV-1 recognition of CCR5 co-receptors in CD4+ human T cells by introducing NHEJ in the endogenous *CCR5* through the employment of ZFNs, effectively mimicking the naturally occurring homozygous Δ32 mutation that normally protects an individual from certain HIV-1 infections [83]. A follow-up study was performed, where autologous *CCR5*-modified CD4+ human T cells were introduced to HIV-positive patients and resulted in a selective advantage, thereby reducing the viral load within these patients [84]. These investigators have now been approved to use CRISPR/Cas9-RGNs in T cells derived from cancer patients in order to edit these cells, enabling the cells to target and destroy the cancer [85]. The first clinical trials using CRISPR/Cas9-RGNs to create a PD-1 knock-out in T cells has been approved for the treatment of muscle-invasive bladder cancer, castration resistant prostate cancer, metastatic renal cancer, and metastatic non-small cell lung cancer, and phase I clinical trials commenced in 2016 [86].

### 5.2. Off-Targeting Risks: CRISPR/Cas9 Mechanistic Hurdles

ZFNs, as used in the studies by Perez et al. [83] and Tebas and colleagues [83], are recognized as relatively low-risk for causing off-target mutations when SSB-inducing nickase domains are employed [87]. This drives the cellular repair machinery to repair the break using a region of homology, thus eliminating the risk of unwarranted DNA modifications at off-target sites [87]. In stark contrast, CRISPR/Cas9-RGNs have been critiqued for the number of genome locations that, depending on the construction of the gRNA and version of Cas9 protein, are potential off-targeting candidates [88,89,90]. However, there is also a CRISPR-Cas9 counter-part, CRISPR-D10A Cas9, which has single-stranded nickase activity, akin to ZFN nickases. CRISPR-D10A Cas9 results in no off-target activity, unlike Cas9 alone [91,92]. If Cas9 cleavage is triggered in an unintended location, chromosomal aberrations [93], the down-regulation of a tumor-suppressor gene or the up-regulation of an oncogene [94], or an unforeseen deleterious mutation in a housekeeping gene [95] could be realized post-reintroduction of the edited cells to a patient, where all such events could lead to the establishment of unregulated tumor growth. Off-target effects have been shown to occur when five mismatches exist between the gRNA and the target site [88], but more recently a study by Wang and colleagues identified an off-target site where the target sequence (within the human *SH2D1A* gene) differed by 13 bases to the gRNA, but this may have been due to single-nucleotide skipping establishing only a single mismatch [91]. A number of studies have shown that mismatches between the gRNA and target site when using CRISPR/Cas9-RGNs may be tolerable at the PAM-distal, but not the PAM-proximal end of the crRNA-portion of the gRNA [74,96,97,98]; however, Fu et al. [88] found that mismatches throughout the length of the gRNA are well tolerated when targeting multiple sites on the EGFP reporter gene within human cells (see Figure 1 for PAM-proximal/PAM-distal orientations). Fu and colleagues also targeted four endogenous human genes, *VEGFA*, *EMX1*, *RNF2*, and *FANCF* and concluded that *VEGFA* was highly susceptible to off-target mutations. Some gRNAs used to target *VEGFA* in this study differed in sequence at the PAM-proximal end, and still led to mutations in the protein-coding region of the *VEGFA* gene [88]. Others have also noted a dosage-dependent relationship between Cas9:gRNA and off-target activity [91], where a reduction in the concentration or Cas9:gRNA complexes transfected into the cells greatly increases the cleavage specificity [90]. These findings indicate that certain aspects of off-targeting activity have yet to be defined, with regards to the mismatch frequency and specific distribution between target sequence and gRNA, including investigations into the tolerance of specific RNA:DNA base-pairing interactions [91]. This corroborates a need of vigilance when utilizing CRISPR/Cas9-RGNs in vivo and suggests that differing regions of the human genome may be more susceptible to off-target effects of CRISPR/Cas9-RGNs than others.

### 5.3. Suppression of NHEJ and Induction of HDR

In addition to controlling off-target activity in CRISPR/Cas9-RGNs, the overall efficiency of the HDR pathway must be optimized to ensure precise genetic change at DNA sites of interest. One method currently being investigated is the suppression of NHEJ in cells targeted for gene editing [99,100]. Li et al. discovered a deficiency in NHEJ activity in *HMGA2*-expressing cells and suggested the overexpression of *HMGA2* as an NHEJ-suppressor; however, an underlying oncogenic nature of *HMGA2* prevents the overexpression of this gene as a method for inducing HDR over NHEJ for clinical applications [99]. In a study by Chu and colleagues, a main proponent in the NHEJ pathway, DNA Ligase IV, was silenced in order to assess the effect of this silencing event on CRISPR/Cas9-induced DSB repair [100]. Chu et al. determined that DSB repair is in favor of HDR, and that NHEJ is suppressed when DNA Ligase IV has been silenced, thus providing a tangible method for enhancing precise editing events in human cells [100]. Furthermore, recent investigations have been concerned with increasing HDR activity by implementing modifications to the exogenous correction template [81,101]. In cases where mutations occur in the heterozygous form, use of an exogenous correction template may not be necessary due to the efficiency of HDR when an endogenous region of homology exists, namely the unaffected allele [63]. This endogenously induced HDR cannot be employed in cases where mutations exist in the homozygous form, making correction templates necessary for these cases. In previous studies, the correction template utilized has varied in multiple aspects, including symmetry around the Cas9 cut site [63,81,102]. In a thorough study by Richardson and colleagues, a number of single-stranded donor templates were analyzed for HDR efficiency [81]. Richardson et al. identified a process at the Cas9 cut site whereby the PAM-distal strand is available for correction template interaction [81]. Upon this discovery, various designs of correction templates with respect to the Cas9 cut site were posited, and further investigation led to the conclusion that asymmetric donor DNA (36 bp PAM-distal, 91 bp PAM-proximal), complimentary to the non-target strand, yields the highest HDR frequencies [81]. Going forward, similar exogenous correction template designs should be verified in conjunction with CRISPR/Cas9-RGNs for the correction of deleterious mutations in LSD patient-derived cell lines.

### 5.4. CRISPR/Cas9 Application to LSDs

Although underlying disease-specific challenges must be evaluated and addressed accordingly, this previously discussed CRISPR/Cas9 system in conjunction with iPSCs is a practical method of treatment for the majority of LSDs. A number of LSDs qualify as candidates for gene editing due to the specific disease (a) resulting from mutations in a single gene; (b) having mechanisms in place whereby supplemental lysosomal enzymes can be taken up and utilized in target tissues; and (c) having limited available therapies. For example, Sanfilippo B syndrome is a rare LSD caused by the mutations in *N*-acetyl glucosaminidase (*NAGLU*) and characterized by progressive neurodegeneration with no current effective treatment [103]. Since Sanfilippo B syndrome is monogenic, and has been shown to utilize the aforementioned cross-correction pathways, CRISPR/Cas9-RGNs could be used to target private mutations in *NAGLU* using patient-derived iPSCs, followed by differentiation to neural progenitors and intracerebral transplantation back to the patient for disease amelioration [60]. Pompe disease is caused by mutations in the gene encoding the lysosomal hydrolase acid-alpha glucosidase and is characterized by progressive myopathy [104]. Patients with Pompe disease can benefit from the ERT Myozyme^®^; however, ERT (as discussed in Section 1.2) is an expensive, lifelong treatment. HSCT using CRISPR/Cas9 corrected HSCs derived from the patients themselves would allow for alleviation of the disease symptoms in patients with Pompe disease, for HSCT has been described as a successful regenerative therapy in mouse models by van Til et al. [105]. A third LSD that is a potentially suitable target for this technique is Niemann-Pick A disease due to the monogenic nature and successful downstream applications of neural progenitors via intracerebral transplantation in mouse models [60]. Although not explicitly discussed here, many other LSDs are justifiable targets for this aforementioned regenerative therapy using CRISPR/Cas9-RGNs and iPSCs (see Table 1 for examples).

### 5.5. LSD-Specific Gene Editing Hurdles

In cases where many disease-causing private mutations exist for a particular disease, CRISPR/Cas9-RGNs must be individually designed and tested for that patient’s genotype. This specificity is exacerbated when genotypes exist in the compound heterozygous form, thus calling for multiple applications of CRISPR/Cas9-RGNs to target and correct the underlying mutations or more complicated CRISPR/Cas designs, such as the *piggyBac* transposon method [61]. Correction of both existing mutations may not be necessary, however, in cases where the disease threshold is low and requires only a small percentage of normal enzyme activity, such as in Hurler syndrome where patients with as little as 0.13% normal alpha-l-iduronidase activity results in a mild phenotypic form [25]. In GD, the most common LSD, the use of CRISPR/Cas9 gene editing is particularly challenging, for *GBA1* implicated in GD shares 96% sequence similarity with a pseudogene (*GBAP1*) 16 kb downstream [106]. In the reverse direction, the sequence of *GBAP1* constitutes part of the coding sequence of metaxin 1 (*MTX1*), an essential gene that encodes a mitochondrial outer membrane protein. This further complicates the use of CRISPR/Cas9-RGNs for targeting of *GBA1* because of the high probability of also targeting *GBAP1/MTX*, which could result in a decreased efficiency of *GBA1* editing and disruption in the *MTX1* sequence [107,108]. In addition to the complexities of gene editing methods due to diversity of disease-causing mutations, heterozygosity and other aforementioned hurdles, downstream applications of mutation-corrected cells must still be addressed. Tay Sachs disease (TSD), an LSD resulting from mutations in *HexA*, the gene that encodes hexosaminidase A, initially seems to be a suitable candidate for CRISPR/Cas9 gene editing based on the monogenic nature of the disease. However, studies that have shown variability in efficacy of cross-correction mechanisms in this disease pathway indicate that other downstream methods may need to be developed and employed to overcome the disease state in vivo [109,110,111,112]. In addition, varied reversibility of disease progression in TSD patients may be seen post-treatment with CRISPR/Cas9 edited cells, depending on the age and disease severity at the time of treatment [113]. These hurdles must also be taken into consideration when evaluating the suitability of gene therapies for other LSDs.

## 6. Conclusions

In conclusion, CRISPR/Cas9 is an effective and relatively inexpensive gene editing technique that shows promise as a novel treatment option for genetic disease where currently available treatment options fall short. This system has been proven to correct mutations in vitro associated with diseases, such as β-thalassemia and cystic fibrosis, and is currently being used in vivo through phase I clinical trials for cancer therapy and the reduction of viral load in patients with HIV. Many LSDs arise from mutations in a single gene, have mechanisms in place for the transfer of lysosomal enzymes from edited cells post-transplant and currently lack alternative treatment options. The use of mutation correction in iPSCs through CRISPR/Cas9 gene editing and subsequent differentiation of these cells to progenitors for engraftment can overcome the lack of therapeutic options currently available for patients with LSDs. Although there are underlying issues in this gene editing system that must be considered and overcome, novel methods to improve this system are rapidly emerging.

## Figures and Tables

**Table 1:** Current treatment options available for a number of lysosomal storage diseases (LSDs). Potential use of CRISPR/Cas9 for correction of disease causing mutations is indicated for each LSD listed.

LSD		Gene Affected	Current Treatment Options *	Example Drugs Available (Drug, *Company*)	Overall CRISPR/Cas9 Suitability (+/−)	References
Gaucher Disease (GD)		*GBA1*	ERT; SRT; PCT	Ceredase^®^, *Genzyme* (Cambridge, MA, USA); Cerdelga^®^, *Genzyme* (Cambridge, MA, USA); Mucoslovan^®^; *Boehringer Ingelheim* (Biberach, Germany)	+ ^†^	[23,33,34,35,36,37]
Sanfilippo Syndrome (MPS III)	A	*SGSH*	SRT	Genistein ^‡^	+	
	B	*Naglu*				[38]
	C	*HGSNAT*				
	D	*GNS*				
Fabry		*alpha-Gal A*	ERT	Fabrazyme^®^, *Genezyme* (Cambridge, MA, USA)	+	[39,40,41]
Tay Sachs		*HexA*	-	-	−	[42]
I-cell disease		*GNPTAB*	-	-	− ^§^	[43]
Niemann-Pick C Disease (NPC)		*NPC1 or NPC2*	SRT	Zavesca^®^, *Actelion* (Allschwil, Switzerland)	+	[19,44,45,46]
MPS I		*IDUA*	ERT	Aldurazyme^®^, *Genzyme* (Cambridge, MA, USA)	+	[47]
MPS II		*IDS*	ERT	Hunterase^®^, *CytoBioteck* (Bogota, Colombia)	+	[48]
MPS VI		*ARSB*	ERT	Naglazyme^®^, *Biomarin* (San Rafael, CA, USA)	+	[49]
Pompe disease		*GAA*	ERT	Myozyme^®^ *Genzyme* (Cambridge, MA, USA)	+	[50,51]
Niemann-Pick A disease		*SMPD1*	-	-	+	[52,53]

* Only enzyme replacement therapy (ERT), substrate reduction therapy (SRT), pharmacological chaperone therapy (PCT) as current treatment options are indicated; ^†^ refer to Section 5.4 for *GBAP1* complications; ^‡^ genistein, a naturally-occurring isoflavone, has been shown to reduce urinary secretions of glycosaminoglycans, but has yet to be tested at higher, clinically relevant doses for SRT in MPS III patients [38]; ^§^ although mutations in GlcNAc phosphotransferase are suitable targets for CRISPR/Cas9 gene editing, lack of disease amelioration post-hematopoietic stem cell transplantation (HSCT) indicates that applicability of gene therapy approaches may be limited in these patients [29].
